# Obese patients experience more severe OSA than non-obese patients

**DOI:** 10.1097/MD.0000000000031039

**Published:** 2022-10-14

**Authors:** Shih-Chun Hsing, Chu-Chieh Chen, Shi-Hao Huang, Yao-Ching Huang, Ren-Jei Chung, Chi-Hsiang Chung, Wu-Chien Chien, Chien-An Sun, Shu-Min Huang, Pi-Ching Yu, Chun-Hsien Chiang, Shih-En Tang

**Affiliations:** a Center for Healthcare Quality Management, Cheng Hsin General Hospital, Taipei, Taiwan; b Department of Health Care Management, College of Health Technology, National Taipei University of Nursing and Health Sciences, Taipei, Taiwan; c Department of Chemical Engineering and Biotechnology, National Taipei University of Technology, Taipei Tech, Taiwan; d School of Public Health, National Defense Medical Center, Taipei, Taiwan; e Department of Medical Research, Tri-Service General Hospital, Taipei, Taiwan; f Graduate Institute of Life Sciences, National Defense Medical Center, Taipei, Taiwan; g Taiwanese Injury Prevention and Safety Promotion Association (TIPSPA), Taipei, Taiwan; h Department of Public Health, College of Medicine, Fu-Jen Catholic University, New Taipei City, Taiwan; i Big Data Center, College of Medicine, Fu-Jen Catholic University, New Taipei City, Taiwan; j Deaprtment of Infection Control, Taipei Medical University Hospital, Taipei City, Taiwan; k Cardiovascular Intensive Care Unit, Department of Critical Care Medicine, Far-Eastern Memorial Hospital, New Taipei City, Taiwan; l Graduate Institute of Medicine, National Defense Medical Center, Taipei, Taiwan; m Department of Cardiovascular Medicine, Far-Eastern Memorial Hospital, New Taipei City, Taiwan; n Department of Internal Medicine, Division of Pulmonary and Critical Care Medicine, Tri-Service General Hospital, National Defense Medical Center, Taipei, Taiwan; o Institute of Aerospace and Undersea Medicine, National Defense Medical Center, Taipei, Taiwan.

**Keywords:** National Health Insurance Research Database (NHIRD), nested case–control study, obesity, obstructive sleep apnea (OSA)

## Abstract

To investigate whether previous exposure to obstructive sleep apnea (OSA) increases the risk of obesity in obese and nonobese patients. We identified 24,363 obese patients diagnosed between January 1, 2000, and December 31, 2015, in the Taiwan Longitudinal Health Insurance Database (LHID) 2005 National Health Insurance Research Database; 97,452 sex-, age- and index date-matched nonobese patients were identified from the same database. This study is based on the ninth edition of the International Classification of Sleep Disorders. Multiple logistic regression was used to analyze the previous exposure of obese patients to OSA. *P* < .05 was considered significant. The average age of 121,815 patients was 44.30 ± 15.64 years old; 42.77% were males, and 57.23% were females. Obese patients were more likely to be exposed to OSA than nonobese patients (adjusted odds ratio [AOR] = 2.927, 95% CI = 1.878–4.194, *P* < .001), and the more recent the exposure period was, the more severely obese the patient, with a dose-response effect (OSA exposure < 1 year, AOR = 3.895; OSA exposure $\underset{-}{\geq }$1 year, <5 years, AOR = 2.933; OSA exposure $\underset{-}{\geq }$5 years, AOR = 2.486). The probability of OSA exposure in obese patients was 2.927 times that in nonobese patients, and the longer the exposure duration was, the more severe the obesity situation, with a dose-response effect (OSA exposure < 1 year, AOR = 2.251; OSA exposure $\underset{-}{\geq }$1 year, <5 years, AOR = 2.986; OSA exposure $\underset{-}{\geq }$5 years, AOR = 3.452). The risk of obesity in subjects with OSA was found to be significantly higher in this nested case–control study; in particular, a longer exposure to OSA was associated with a higher likelihood of obesity, with a dose-response effect.

## 1. Introduction

Obstructive sleep apnea (OSA) is the most common sleep-related breathing disorder. It is characterized by repeated episodes of complete or partial obstruction of the upper airway, resulting in decreased or no breathing during sleep. These events are called “apnea” when breathing is completely or nearly completely stopped or “hypopnea” when breathing is reduced. In either case, blood oxygen saturation will drop, and sleep interruption will occur, or both. A high frequency of apnea or hypopnea during sleep may interfere with restorative sleep, and sleep apnea and blood oxygenation disorders together lead to negative effects on health and quality of life.^[1]^ The terms obstructive sleep apnea syndrome or obstructive sleep apnea hypopnea syndrome can be used to refer to OSA when it is associated with symptoms during the day (e.g., excessive daytime sleepiness, reduced cognitive function).^[2,3]^

OSA is classified as a sleep-related respiratory system disease and is divided into two categories (adult OSA and pediatric OSA) in the third edition of the International Classification of Sleep Disorders.^[4]^ The difference between OSA and central sleep apnea is that central sleep apnea is characterized by reduced or stopped breathing due to reduced effort rather than upper airway obstruction.^[5]^ It is necessary to evaluate respiration to correctly classify apnea as obstructive, considering the specificity of muscle activity in the absence of airflow and whether respiration continues or increases.^[6,7]^ When hypopnea and apnea coexist, it is classified as obstructive sleep apnea hypopnea (OSAH), and when it is associated with daytime sleepiness and other daytime symptoms, it is called obstructive sleep apnea hypopnea syndrome.^[8]^

Obesity is a worldwide epidemic, and its prevalence is increasing in most Western societies and developing countries. If this trend continues, by 2025, the global obesity rate for men will reach 18%, and for women, it will exceed 21%.^[9]^ In addition, obesity (depending on the degree, duration and distribution of excess body weight/fat tissue) will gradually cause and/or aggravate various complications, including type 2 diabetes, hypertension, dyslipidemia, cardiovascular disease, nonalcoholic fatty liver disease, reproductive dysfunction, abnormal breathing, and mental illness, and even increase the risk of certain types of cancer.^[9]^ World Health Organization pointed out that “obesity is a chronic disease” and called for attention to be paid to the health hazards of obesity. According to 2016 World Health Organization data, more than 1.9 billion adults 18 years and older were overweight. Of these, over 650 million were obese.^[10]^ There were 7 items related to obesity, including cancer, heart disease, cerebrovascular disease, diabetes, hypertensive disease, nephritis, renal syndrome and nephropathy, chronic liver disease and cirrhosis, among the top ten causes of death among Taiwanese individuals in 2017.^[11]^ The National Health Administration of the Ministry of Health and Welfare released the results of the latest survey, showing that the rate of obesity among adults over 18 years of age has risen from 38% in 2009 to 43.9% in 2018.^[12]^ Compared with people of healthy weight, obese people have more than three times the risk of diabetes, metabolic syndrome and dyslipidemia and two times the risk of hypertension, cardiovascular disease, knee arthritis and gout.^[12]^

Some risk factors, including obesity, male sex, age, and genetic factors, are related to an increased prevalence of OSA in the general population.^[13]^ Among them, obesity is one of the strongest risk factors for sleep apnea.^[14]^ Mild to moderate obesity has been associated with a significant increase in the prevalence of sleep apnea.^[15]^ OSA can adversely affect multiple organs and systems, especially those related to cerebrovascular disease. Several diseases, such as hypertension, insulin resistance, systemic inflammation, visceral fat deposition and dyslipidemia, are also present in other diseases closely related to OSA, such as obesity and reduced sleep duration.^[16]^ Current longitudinal observational studies investigating the relationship between OSA and obesity are limited. Based on previous studies, we hypothesized that obesity is associated with OSA. Therefore, we used the National Health Insurance Research Database (NHIRD) of the Ministry of Health and Welfare to investigate whether OSA increases the subsequent risk of obesity.

## 2. Methods

### 2.1. Data source

**Figure 1.**
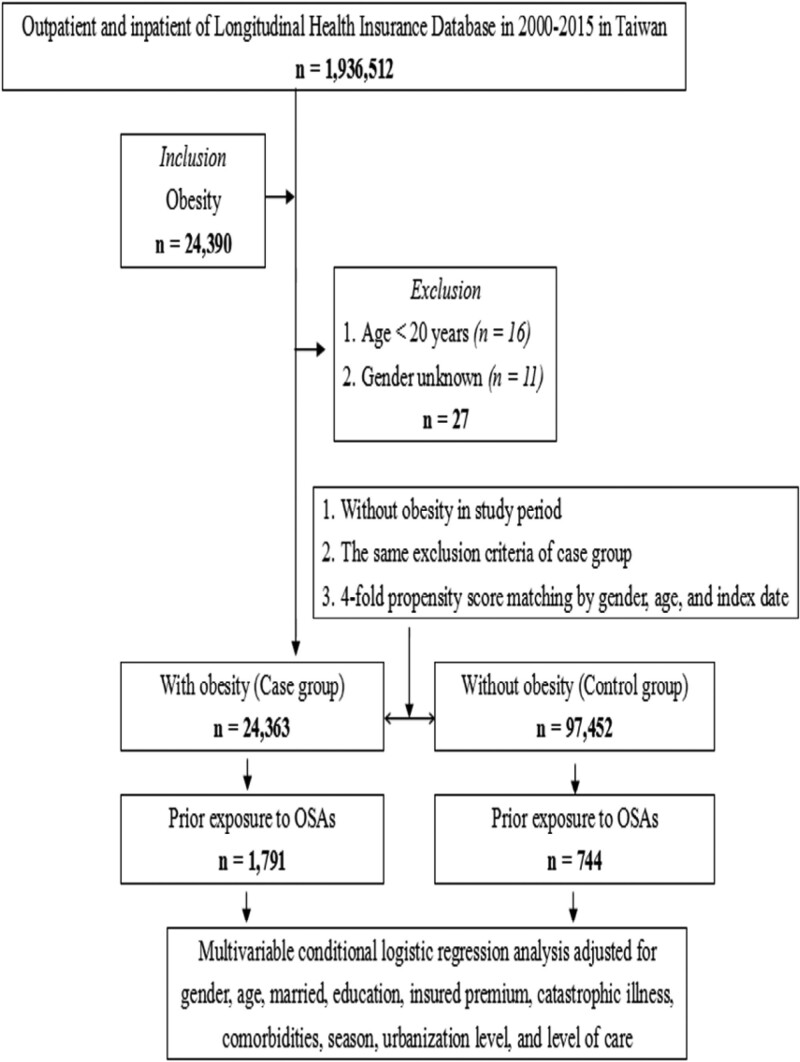
Flowchart of the study design (case–control study) using the National Health Insurance Research Database in Taiwan. OSA = obstructive sleep apnea.

Taiwan’s National Health Insurance Plan launched the single payer system on March 1, 1995. As of 2017, 99.9% of Taiwan’s population was enrolled. In this study, data were collected from the 2005 Longitudinal Health Insurance Database (LHID2005), which is part of the National Health Insurance Research Database, and 2000,000 people were randomly selected from the entire population. The National Institutes of Health encrypted all personal identities before issuing LHID2005 to protect the privacy of patients. In the LHID2005 file, the disease diagnosis code is based on the “International Classification of Diseases, Ninth Revision, Clinical Modification” (ICD-9-CM).^[17]^ The flowchart of study design (nested case–control study) from the National Health Insurance Research Database in Taiwan (Fig. 1). The Ethical Review Board of the Tri-Service General Hospital of the National Defense Medical Center (TSGHIRB No. B-109-39) approved this study.

### 2.2. Determination of cases and controls

If the patient was diagnosed with obesity according to International Classification of Diseases, Ninth Revision, Clinical Modification (ICD-9-CM) (ICD-9-CM code 178) between 2000 and 2015, the patient was included in the obesity case group. The control group comprised nonobese patients; indexed dates were grouped by sex and age, and the ratio was 1:4.

### 2.3. Identifying OSA, obesity, and comorbidities

The risk factor discussed in this study is OSA, and the definition criteria are that at least three outpatient diagnoses must be made with ICD-9 codes. These codes include 780.51, 780.53, and 780.57 (sleep apnea syndrome) from 2000 to 2015.

The obesity outcomes included overweight, obesity and other hyperalimentation (ICD-9-CM code 278), overweight and obesity (ICD-9 CM code 278.0), morbid obesity (ICD-9-CM code 278.01), overweight (ICD-9-CM code 278.02), and obesity hypoventilation syndrome (ICD-9-CM code 278.03).

The comorbidities evaluated in this study included diabetes (ICD-9-CM 250), hypertension (ICD-9-CM 401-405), hyperlipidemia (ICD-9- CM 272.4), CAD (ICD-9-CM 414.01), stroke (ICD-9-CM 430-438), CHF (ICD-9-CM 428.0), chronic obstructive pulmonary disease (ICD-9-CM 490-496), chronic kidney disease (ICD-9-CM 585), liver cirrhosis (ICD-9-CM 571.5), tumor (ICD-9-CM 199), anxiety (ICD-9-CM 300.00), and depression (ICD-9-CM 296.2–296.3, 300.4, 311).

### 2.4. Statistical analysis

Descriptive statistical information was used for characteristic information, including percentages, means and standard deviations. Chi-square tests and *t* tests were used to evaluate the distribution of categorical and continuous variables between cases and controls. Conditional logistic regression analyses were performed to evaluate the risks of obesity associated with OSA (with and without) adjusting for age, sex, education, insurance premium, comorbidities, Charlson Comorbidity Index, season, location, urbanization level, and level of care. Factors related to obesity and the first OSA exposure before obesity diagnosis were examined by using conditional logistic regression. All analyses were performed using SPSS version 22 (IBM, Armonk, NY). *P* < .05 was considered significant.

### 2.5. Patient and public involvement

The study was secondary data analysis. The data from the nationwide, population-based, nested case–control study described herein were obtained from the National Health Insurance Research Database in Taiwan. The requirement for informed consent from each patient was waived.

## 3. Results

### 3.1. Demographic data

In Table 1, the average age of 121,815 patients was 44.30 ± 15.64 years, of which 42.77% were males, and 57.23% were females. There were 24,363 obese patients, and there were approximately 97,452 nonobese controls. Compared with those in the control group, those in the obesity group had a higher prevalence of comorbidities. Charlson Comorbidity Index, season, location, urbanization level, and level of care were significantly different between the case and control groups.

**Table 1:** Characteristics of study patients.

Obesity	Total		Cases		Controls		*P* value
Variables	n	%	n	%	n	%	
Total	121,815		24,363	20.00	97,452	80.00	
OSA exposure							
Without	119,280	97.92	22,572	92.65	96,708	99.24	<.001
With	2535	2.08	1791	7.35	744	0.76	
Sex							
Male	52,105	42.77	10,421	42.77	41,684	42.77	.999
Female	69,710	57.23	13,942	57.23	55,768	57.23	
Age (yr)	44.30 ± 15.64		44.25 ± 15.53		44.31 ± 15.67		.592
Age group (yr)							
20–44	74,135	47.48	14,827	47.48	59,308	47.48	.999
45–64	34,330	21.99	6866	21.99	27,464	21.99	
≥65	47,680	30.54	9536	30.54	38,144	30.54	
Married							
No	55,634	45.67	11,375	46.69	44,259	45.42	<.001
Yes	66,181	54.33	12,988	53.31	53,193	54.58	
Comorbidities							
CCI_R	0.05 ± 0.29		0.07 ± 0.38		0.05 ± 0.26		<.001
Season							
Spring (Mar–May)	28,626	23.50	6110	25.08	22,516	23.10	<.001
Summer (Jun–Aug)	31,018	25.46	6646	27.28	24,372	25.01	
Autumn (Sep–Nov)	33,587	27.57	6201	25.45	27,386	28.10	
Winter (Dec–Feb)	28,584	23.47	5406	22.19	23,178	23.78	
Location							
Northern Taiwan	53,628	44.02	13,166	54.04	40,462	41.52	<.001
Middle Taiwan	32,511	26.69	5135	21.08	27,376	28.09	
Southern Taiwan	29,075	23.87	4760	19.54	24,315	24.95	
Eastern Taiwan	6185	5.08	1250	5.13	4935	5.06	
Outer islands	416	0.34	52	0.21	364	0.37	
Urbanization level							
1 (The highest)	40,015	32.85	8825	36.22	31,190	32.01	<.001
2 (Second)	52,239	42.88	9839	40.39	42,400	43.51	
3 (Third)	10,620	8.72	1644	6.75	8976	9.21	
4 (The lowest)	18,941	15.55	4055	16.64	14,886	15.28	
Level of care							
Hospital center	40,928	33.60	9203	37.77	31,725	32.55	<.001
Regional hospital	55,737	45.76	12,296	50.47	43,441	44.58	
Local hospital	25,150	20.65	2864	11.76	22,286	22.87	

*P*: Chi-square/Fisher exact test for categorical variables and *t* test for continuous variables.

CCI = Charlson Comorbidity Index, OSA = obstructive sleep apnea.

### 3.2. Logistic regression of obesity variables

Table 2 shows a significantly higher risk of obesity in the OSA group than in the control group (adjusted odds ratio [AOR] = 2.927, 95% CI = 1.878–4.194). Male patients were 0.759 times more likely than female patients to be obese (AOR = 0.759, 95% CI = 0.735–0.783). Among the age groups, there was a significantly lower risk of obesity for subjects aged 45 to 64 years old or ≥65 years old (AOR = 0.595, 95% CI = 0.572–0.619; AOR = 0.417, 95% CI = 0.394–0.441). Summer and Winter were associated with significantly lower obesity risks (AOR = 0.833, 95% CI = 0.799–0.869; AOR = 0.866, 95% CI = 0.829–0.905, respectively).

**Table 2:** Logistic regression of obesity variables.

Variables	AOR	95% CI	*P* value
OSA			
Without	Reference		
With	2.927	1.878–4.194	<.001
Sex			
Male	0.759	0.735–0.783	<.001
Female	Reference		
Age group (yr)			
20–44	Reference		
45–64	0.595	0.572–0.619	<.001
$\underset{-}{\geq }$65	0.417	0.394–0.441	<.001
Season			
Spring	Reference		
Summer	0.833	0.799–0.869	<.001
Autumn	0.966	0.955–1.039	.858
Winter	0.863	0.829–0.905	<.001

Variables listed in the table.

AOR = adjusted odds ratio, CI = confidence interval, OSA = obstructive sleep apnea.

### 3.3. Using logistic regression to stratify the obesity factors

In Table 3, we observed that the risk of obesity was higher in subjects with OSA than in controls (AOR = 2.927, 95% CI = 1.878–4.194). Female patients with OSA were 4.384 times more likely than controls to be obese (AOR = 4.384, 95% CI = 2.813–6.281). Among the age groups, there was a significantly higher risk of obesity with OSA for subjects aged 20 to 44 years than for controls (AOR = 8.732, 95% CI = 5.603–12.512). Spring OSA diagnoses were associated with significantly higher obesity risks than controls (AOR = 3.032, 95% CI = 1.946–4.345) by conditional logistic regression analyses.

**Table 3:** Relationship between factors affecting obesity and OSA according to logistic regression.

Group	With OSA vs without OSA (reference)		
Stratification	AOR	95% CI	*P* value
Overall	2.927	1.878–4.494	<.001
Sex			
Male	2.445	1.569–3.503	<.001
Female	4.384	2.813–6.281	<.001
Age group (yr)			
20–44	8.732	5.603–12.512	<.001
45–64	3.127	2.007–4.481	<.001
≥65	2.463	1.580–3.529	<.001
Season			
Spring	3.032	1.946–4.345	<.001
Summer	2.718	1.744–3.894	<.001
Autumn	3.015	1.935–4.321	<.001
Winter	2.945	1.890–4.220	<.001

Adjusted for the variables listed in Table 3.

AOR = adjusted odds ratio, CI = confidence interval, OSA = obstructive sleep apnea.

### 3.4. Using logistic regression to analyze obesity factors by duration of obstructive sleep apnea exposure

Table 4 shows that obese patients were more likely to be exposed to OSA than nonobese patients (AOR = 2.927, 95% CI = 1.878–4.194), and the more recent the exposure, the more severe the situation was, with a dose-response effect (OSA exposure <1 year, AOR = 3.895; OSA exposure ≥1 year, <5 years, AOR = 2.933; OSA exposure ≥5 years, AOR = 2.486).

**Table 4:** Relationship between factors affecting obesity and OSA exposure according to conditional logistic regression.

Time between first OSA exposure and the last exposure before obesity diagnosis	With OSA vs without OSA (reference)		
	AOR	95% CI	*P* value
Overall	2.927	1.878–4.194	<.001
<1 yr	3.895	2.385–5.796	<.001
≥1 yr, <5 yr	2.933	1.816–4.025	<.001
≥5 yr	2.486	1.533–3.287	<.001

Adjusted for the variables listed in Table 2.

AOR = adjusted odds ratio, CI = confidence interval, OSA = obstructive sleep apnea.

In addition, Table 5 shows that the probability of exposure to OSA in obese patients was 2.927 times that in nonobese patients (AOR = 2.927, 95% CI = 1.878–4.194), and the longer the exposure duration was, the more severe the obesity situation, with a dose-response effect (OSA exposure <1 year, AOR = 2.251; OSA exposure ≥1 year, <5 years, AOR = 2.986; OSA exposure ≥5 years, AOR = 3.452).

**Table 5:** Relationship between factors affecting obesity and duration of OSA exposure according to conditional logistic regression.

Duration of OSA exposure prior to obesity diagnosis	With OSA vs without OSA (reference)		
	AOR	95% CI	*P* value
Overall	2.927	1.878–4.194	<.001
<1 yr	2.251	1.503–3.970	<.001
≥1 yr, <5 yr	2.986	1.911–4.283	<.001
≥5 yr	3.452	2.445–4.996	<.001

Adjusted for the variables listed in Table 2.

AOR = adjusted odds ratio, CI = confidence interval, OSA = obstructive sleep apnea.

## 4. Discussion

The results of this study found that female patients have a significantly higher risk of obesity than male patients. This is consistent with the study of Kanter and Caballero^[18]^ The obesity rates of men and women vary greatly within and between countries, and overall, men have lower rates of obesity than women.^[18]^ Compared with patients aged 45 to 64 or ≥65, patients aged 20 to 44 have a significantly higher risk of obesity. A possible reason is that young people have increased their food intake, and their lifestyles have become more sedentary, which has greatly increased the prevalence of obesity and metabolic diseases; however, there may be unknown factors affecting this result.^[19]^ In addition, compared with spring, in summer and winter, the risk of obesity is much lower, which is consistent with the results of the Ma et al^[20]^ study. The relationship between seasonal changes in body weight and seasonal changes in food intake and physical activity, and in particular the relationship between the relative importance of these factors in determining weight changes, has not been fully studied.^[20]^ OSA increases the risk of comorbidities, such as systemic hypertension, diabetes, chronic kidney disease, chronic obstructive pulmonary disease, asthma, cardiovascular events and/or death, arrhythmias, stroke and cancer. The results of Bonsignore et al^[21]^ are similar to ours. In addition, patients with mild OSA who had a 10% weight gain increased their risk of OSA progression by 6 times, and the same weight loss could lead to a 20% improvement in OSA severity.^[22]^ Although there is evidence that obesity and visceral obesity may cause OSA and that weight loss improves OSA symptoms, recent studies have shown that OSA may cause obesity.^[23,24]^ There is a linear relationship between obesity and OSA. In obese people, fat deposits in the upper respiratory tract narrow the airways. Muscle activity in this area is reduced, leading to hypoxia and apnea episodes, which eventually lead to sleep apnea.^[25]^

The relationship between OSA and obesity is complex, and current research has proposed a possible mechanism for the link between OSA and obesity. Obesity may worsen OSA because of fat deposits in specific areas. Fat deposits in the tissues surrounding the upper respiratory tract seem to result in a smaller lumen and increase collapsibility of the upper respiratory tract, predisposing patients to apnea.^[26,27]^ In addition, fat deposits around the chest (truncal obesity) reduce chest compliance and functional residual capacity and can increase oxygen demand.^[28]^ Visceral obesity is common in OSA patients.^[29]^ Several studies believe that excessive food intake will increase the risk of obesity in short-duration sleepers, where a short sleep duration is related to changes in the hormones responsible for hunger and appetite control (especially leptin and ghrelin).^[30]^ Leptin is a participatory energy intake and metabolic hormone. High leptin levels are common in obstructive sleep apnea syndrome patients. The level of the hormone leptin and the severity of OSA are related.^[31]^ Another study showed that obesity and obstructive sleep apnea patients had elevated levels of the hormone leptin, and leptin is proportional to the severity of metabolic syndrome. In OSA patients, serum leptin levels were 50% higher than those in the control group.^[32]^ The abovementioned pathophysiological factors may explain the association between OSA and obesity shown in this study. Our research results show that the prevalence of obesity in those exposed to OSA is 2.927 times that in nonobese patients. The more recent the exposure and the longer the exposure duration, the more serious the obesity situation is. Therefore, it is necessary to pay attention to the relationship between the occurrence and duration of OSA and obesity.

This study has several potential limitations. First, the National Health Insurance Research Database does not provide detailed information on aspects such as drinking, smoking, physical activity, and eating behavior, which may lead to confounding. Second, Obesity is classified as morbid obesity in the NHIRD of the Ministry of Health and Welfare and is not classified as overweight, obesity level 1, obesity level 2, and extremely obese. Third, body mass index was not a variable in our study. Finally, although this study was carefully designed and controlled for confounding factors, there may still be biases due to unmeasured or unknown confounding factors (such as the onset of depression, the stage of obesity at the time of diagnosis, and the use of drugs that may affect the outcome). A prospective cohort study is recommended to evaluate the relationship between OSA and obesity.

## 5. Conclusion

This study revealed that *obesity is significantly associated with OSA*. Furthermore, the closeness to the time of the study and the exposure duration were both positively related to the severity of obesity, with a dose–response effect. OSA may be a risk factor for obesity. Health care providers should pay close attention to the association between OSA and the risk of obesity.

## Acknowledgments

We wish to thank Taiwan’s Health and Welfare Data Science Center and the Ministry of Health and Welfare for providing the National Health Insurance Research Database (NHIRD). This study was supported by the Tri-Service General Hospital Research Foundation (TSGH-B-111018), and the sponsor has no role in study design, data collection, and analysis, decision to publish, or preparation of the manuscript.

## Author contributions

**Conceptualization:** Wu-Chien Chien, Shi-Hao Huang, Yao-Ching Huang, Shih-Chun Hsing, Chu-Chieh Chen, Shu-Min Huang, Ren-Jei Chung, Pi-Ching Yu, Chun-Hsien Chiang, Shih-En Tang.

**Formal analysis:** Shih-Chun Hsing, Chu-Chieh Chen, Chien-An Sun, Shi-Hao Huang, Yao-Ching Huang, Shu-Min Huang, Ren-Jei Chung, Pi-Ching Yu, Shih-En Tang.

**Investigation:** Chi-Hsiang Chung, Chien-An Sun, Shih-Chun Hsing, Chu-Chieh Chen, Shi-Hao Huang, Yao-Ching Huang, Shu-Min Huang, Ren-Jei Chung.

**Methodology:** Wu-Chien Chien, Chi-Hsiang Chung, Chien-An Sun, Shi-Hao Huang, Yao-Ching Huang, Shu-Min Huang, Pi-Ching Yu, Chun-Hsien Chiang, Shih-En Tang.

**Project administration:** Wu-Chien Chien, Chi-Hsiang Chung, Shih-Chun Hsing, Chu-Chieh Chen, Shi-Hao Huang, Yao-Ching Huang, Shu-Min Huang.

**Writing – original draft:** Shi-Hao Huang, Yao-Ching Huang, Shih-Chun Hsing, Chu-Chieh Chen, Shu-Min Huang, Ren-Jei Chung, Chun-Hsien Chiang, Shih-En Tang.

**Writing – review & editing:** Wu-Chien Chien, Shih-Chun Hsing, Chu-Chieh Chen, Chien-An Sun, Shi-Hao Huang, Yao-Ching Huang, Shu-Min Huang, Ren-Jei Chung, Pi-Ching Yu, Chun-Hsien Chiang, Shih-En Tang.
